# Functional near-infrared spectroscopy (fNIRS) detects brain changes for apathy and pain in patients with Alzheimer's disease and related dementias: An exploratory study^☆^

**DOI:** 10.1016/j.ynirp.2025.100266

**Published:** 2025-05-29

**Authors:** Allison J. Huff, Juyoung Park, Samuel Montero-Hernandez, Lindsey Park, Chiyoung Lee, Luca Pollonini, Hyochol Ahn

**Affiliations:** a Department of Family and Community Medicine, College of Medicine, The University of Arizona, 655 N Alvernon Way, Tucson, AZ, 85711, USA; b College of Nursing, The University of Arizona, 1305 N Martin Ave, Tucson, AZ, 85721, USA; c School of Computer Science, University of Birmingham, Edgbaston, Birmingham, B15 2TT, UK; d Department of Engineering Technology, Cullen College of Engineering, University of Houston, 4230 Martin Luther King Boulevard. #304, Houston, TX, 77204-4020, USA

**Keywords:** Dementia, Alzheimer's disease, fNIRS, Chronic pain, Brain imaging

## Abstract

Alzheimer's Disease and Related Dementias (ADRD) are degenerative and progressive in nature and are often accompanied by chronic pain and neuropsychiatric symptoms, which can be early signs and aggravators of ADRD. This exploratory study explores the relationship between self-reported pain, neuropsychiatric symptoms, and pain-evoked cortical hemodynamic changes measured using functional near-infrared spectroscopy (fNIRS) in the prefrontal and primary motor and somatosensory brain cortices bilaterally, stratified by high or low cognitive function in individuals with ADRD. This study analyzed baseline data of 40 individuals with mild to moderate ADRD with knee osteoarthritis.

Baseline data from 40 individuals with mild to moderate ADRD and knee osteoarthritis were analyzed. Measures included self-reported pain, depression, and apathy, along with fNIRS-derived cerebral hemodynamic responses to sub-threshold thermal pain stimulation across five brain regions. The study revealed significant negative correlations for oxyhemoglobin and apathy in the right prefrontal cortex associated with low cognitive function (p = .04) and significant positive correlations for oxyhemoglobin and apathy in the right somatosensory region (p = .04) and for oxyhemoglobin and pain in the medial prefrontal cortex (p = .04) associated with higher cognitive function. Study findings suggest that fNIRS may provide valuable biomarkers for apathy and depression in individuals with ADRD and chronic osteoarthritic pain, with differential patterns based on cognitive function, suggesting neuropsychiatric symptoms may manifest differently depending on the patient's cognitive status. Future studies should explore its utility in larger, diverse samples and clinical interventions targeting neuropsychiatric symptoms.

## 1 Introduction

The U.S. population is aging rapidly, with a projected 82 million people over 65 by 2050—a 47 % increase from 2022 (Mather and Scommegna, 2024). This demographic shift has led to a rising prevalence of Alzheimer's Disease and related dementias (ADRD) (Brayne and Miller, 2017; Langa, 2015). Currently, five to seven million Americans are living with ADRD, a number expected to reach 14 million by 2060 (Alzheimer’s Association, 2024; Centers for Disease Control and Prevention, 2019), creating significant healthcare and familial burdens. Over half of these individuals experience comorbidities, such as chronic pain, and neuropsychiatric symptoms (NPS) (Achterberg et al., 2019; Bornier et al., 2023; Tori et al., 2020; Wimo et al., 2023), complicating diagnosis and treatment due to ADRD-related cognitive decline (Smits et al., 2015). Traditional methods for evaluating physical and emotional pain, such as clinical evaluations and self-report surveys, often lack accuracy in individuals with advanced ADRD due to impaired communication and a declined sense of self-awareness (Achterberg et al., 2019; Lichtner et al., 2014). As ADRD progresses, neuropathological changes in regions like the prefrontal cortex (PFC) and somatosensory (S1) cortices further compromise these measures (Achterberg et al., 2019; Cravello et al., 2019; Karunakaran et al., 2021; Ong et al., 2019). Metabolic changes during the progression of ADRD, such as cortical hemodynamic changes and hypometabolism of glucose are known to be present in individuals with mild, moderate, and advanced ADRD, affecting cognitive function (Robba et al., 2023). In fact, similar metabolic brain changes can be present in people with chronic pain and NPS such as depression and apathy, absent cognitive impairment (Falcon et al., 2024; Kim et al., 2021; Ng et al., 2019). The cerebral metabolic changes in individuals with early and progressed stages of dementia are closely linked to changes in pain perception and responses (Cole et al., 2010) and the manifestation of NPS (Ng et al., 2019). Imaging has shown that central sensitization in pain processing pathways impacts cognitive and emotional processing, creating a bi-directional relationship among chronic pain, NPS and cognitive decline (Cao et al., 2019). Emerging techniques, such as functional near-infrared spectroscopy (fNIRS), offer a promising, non-invasive alternative by detecting cortical hemodynamic changes linked to pain and emotional dysregulation (Chen et al., 2020; Fernandez-Rojas et al., 2017; Khan et al., 2024; Lee et al., 2024; Zhang et al., 2022). fNIRS is a portable, cost-effective imaging tool that measures the relative changes in the concentration of oxygenated and deoxygenated hemoglobin (closely linked to neuronal activity through the neurovascular coupling mechanism), with advantages over traditional neuroimaging methods like functional Magnetic Resonance Imaging (fMRI) and Electroencephalogram (EEG), including better temporal resolution than fMRI and higher spatial resolution than EEG, portability, and usability during movement (Crosson et al., 2010; Kuruvilla et al., 2013; Fernandez-Rojas et al., 2017). It is particularly effective in identifying and differentiating neurodegenerative cognitive conditions and monitoring pain-related neuronal activation in cognitively compromised individuals (Atri, 2014; Pless et al., 2023; Koyanagi et al., 2021; Mei et al., 2023; Soucy et al., 2013). By analyzing changes in oxyhemoglobin (HbO) and deoxyhemoglobin (HbR) during brain activity, fNIRS quantifies the hemodynamic response, providing insights into neuronal function and energy metabolism (Chen et al., 2020; Quaresima and Ferrari, 2019). A study by Mei et al. (2023) demonstrated that cortical activation measured using fNIRS differed between four different types of dementia (frontotemporal lobe Dementia, Lewy body dementia, Parkinson's disease dementia, and Alzheimer's Disease) while performing a verbal fluency and working memory task, suggesting fNIRS may be useful in the diagnosis and differentiation of ADRD. In the context of pain research, fNIRS can be used to investigate cortical responses to painful stimuli. Studies using fNIRS have shown changes in hemodynamic responses in regions of the brain associated with pain processing, such as the S1, insula, and PFC, in response to painful stimuli (Montero-Hernandez et al., 2023; Nguyen et al., 2023; Pollonini et al., 2020). This suggests fNIRS’ utility as a non-invasive biomarker for mild cognitive impairment, NPS such as apathy and depression, and chronic pain (Lee et al., 2024; Xie et al., 2024). Additionally, using fNIRS as a neuroimaging technique provides real-time monitoring opportunities for individuals at various stages of the ADRD continuum, which supports medical professionals in developing timely and effective treatment plans, and monitoring the effectiveness of treatments (Xie et al., 2024). These features make fNIRS a valuable tool for improving the understanding of depressive symptoms and apathy, as well as pain, in individuals with ADRD.

In this pilot study, we explored associations between subjective pain assessment and cortical hemodynamic changes using fNIRS in literature-directed brain regions involved in pain sensing and processing, stratified by cognitive function level (high or low) in a clinical sample of individuals with ADRD. We also investigated correlations between NPS of depression and apathy and cortical hemodynamic changes using fNIRS in those same brain regions stratified by cognitive function level.

## 2 Materials and methods

### 2.1 Design

This study analyzed baseline data prior to intervention from a clinical trial pilot study (NCT04457973). This trial was approved by the Institutional Review Board of the participating Institution. The primary study was an experimenter- and participant-blinded, randomized, sham-controlled clinical trial. The aims of the study were to evaluate the preliminary effects of home-based M1-SO applied tDCS on clinical pain in persons with early-stage ADRD (specific aim1); evaluate the preliminary effects of home-based M1-SO applied tDCS on pain-related cortical response in persons with early-stage ADRD (specific aim 2); and evaluate the feasibility and acceptability of home-based M1-SO–applied tDCS for pain management in persons with early stage ADRD (specific aim 3).

### 2.2 Participants

Study participants (n = 40) were recruited by research staff from participating institutions using study flyers distributed at senior centers, clinics, and community events. Table 1 reveals demographic data for the sample. Participants were screened by study personnel for eligibility and consented either in-person or at a scheduled baseline visit. Eligible subjects were between the ages of 50 and 90 years; had a diagnosis of mild-moderate ADRD; suffered from chronic pain (pain >3 on Numeric Rating Scale 0–100) or as per caregiver reports; had a caregiver for at least 10 h a week who was willing to participate in the study; competent in reading and speaking English; and had no plans to change medication during the study. To clarify, participants were screened using a self-report item assessing average pain over the past three months on a 0–10 scale, with a score greater than 3 indicating eligibility. This threshold is consistent with prior research that defines moderate chronic pain as NRS scores above 3 (Breivik et al., 2008). In contrast, clinical pain at baseline was assessed using a 0–100 Numeric Rating Scale (NRS), which asked participants to rate their average pain over the past 24 h. The 0–100 NRS was used to allow for greater sensitivity in capturing pain variability and to support nuanced analysis in the context of chronic osteoarthritic pain in this population (Ngu et al., 2015).

**Table 1:** Participant demographics.

ID	Age	Gender	Race	Education	Marital Status	MoCA-30
101	75	Male	White	Bachelor's	Married	15
102	62	Male	White	HS/GED	Married	19
103	65	Female	White	Doctorate	Married	19
104	75	Female	White	Associates	Widowed	14
105	76	Male	White	Masters	Married	22
106	74	Male	White	Doctorate	Married	20
107	71	Female	White	Masters	Married	4
108	71	Female	White	Bachelor's	Married	26
109	66	Female	Hisp/Lat	Masters	Married	2
110	78	Male	White	Doctorate	Don't know	15
111	77	Male	White	Bachelor's	Married	22
112	90	Female	White	Bachelor's	Widowed	19
113	51	Female	White	HS/GED	Married	23
114	75	Male	White	Masters	Married	14
115	77	Female	Asian	Doctorate	Divorced	15
116	77	Female	White	Masters	Divorced	13
117	86	Male	White	Bachelor's	Married	23
118	80	Female	Asian	HS/GED	Married	15
119	82	Male	White	Doctorate	Married	15
120	77	Female	White	HS/GED	Married	21
121	73	Female	Black/AA	HS/GED	Married	21
122	70	Male	Black/AA	Associates	Married	25
123	55	Female	White	Associates	Refused	22
124	73	Female	White	Doctorate	Married	24
125	67	Female	White	HS/GED	Divorced	14
126	72	Female	White	Masters	Divorced	23
127	72	Female	White	Doctorate	Married	15
128	72	Female	White	Doctorate	Divorced	23
129	75	Female	White	HS/GED	Divorced	22
130	74	Female	White	HS/GED	Married	25
131	78	Male	White	≤HS/No Dip	Married	25
132	75	Female	White	Masters	Widowed	25
133	76	Female	White	Masters	Widowed	23
134	89	Female	White	Associates	Widowed	22
135	70	Female	White	Bachelor's	Married	23
136	64	Female	White	Bachelor's	Divorced	25
137	67	Female	White	Bachelor's	Divorced	22
138	82	Female	White	Associates	Married	22
139	69	Female	White	Bachelor's	Nvr Married	24
140	63	Female	White	HS/GED	Separated	23

Participants were excluded if they were hospitalized for a neuropsychiatric illness in the last 12 months; had a history of brain surgery, brain tumor, seizure, stroke, or intracranial metal implantation; had an active substance use disorder; or had severely diminished cognitive function (i.e., Mini-Mental Status Exam score ≤15, Telephone-Montreal Cognitive Assessment score ≤15). Participants were a mostly homogenous group comprising college-educated (75 %) non-Hispanic white (90 %) females (72 %).

### 2.3 Procedures and data collection

The primary study consisted of six study visits that included an in-person baseline visit, followed by four virtual visits during which participants were remotely monitored while self- or caregiver-administered tDCS, and an in-person final visit. A post-study telephone follow-up was conducted at three months. The current study analyzed data collected only during the baseline visit prior to any tDCS intervention. Once consented, participants completed a questionnaire to collect demographic and medical history details, such as age, sex, height, weight, duration of pain, current and past treatments for pain, comorbid conditions, and current medications. The criteria of mild-to-moderate ADRD were assessed using the Mini Mental Status Exam (MMSE) and the telephone Montreal Cognitive Assessment (t-MoCA) and then verified by the study PI. Other questionnaires were administered: the NeuroPsychiatric Inventory (NPI) to identify behavioral and psychological symptoms and the Numeric Rating System (NRS) to determine baseline pain level. At baseline, each participant was fitted with a cap as part of fNIRS imaging while they underwent subthreshold thermal pain stimulation specific to each individual's previously determined subthreshold level. Subthreshold pain was determined by research staff applying heat pain stimuli to all participants' right arms, and the individualized temperatures were calculated by averaging three trials of heat pain thresholds (the point at which participants first felt the heat becoming uncomfortable on their arm) indicating to the researcher when the pain stimulus to the right arm reached a level of discomfort. The thermal pain stimulation during the trial consisted of three 20-sec low-intensity thermal stimuli blocks (30-sec between blocks) to the right forearm of the participant by using a pain generator (Medoc TSA-II Neurosensory Analyzer, 16 mm × 16 mm thermode) and cortical hemodynamic changes were measured using fNIRS during pain stimulation. Channels covering five brain regions of interest (ROI) were analyzed for this study: 1) right dorsolateral prefrontal cortex (rdlPFC), 2) medial prefrontal cortex (mPFC), 3) left dorsolateral prefrontal cortex (ldlPFC), 4) right somatosensory cortex (rS1), and 5) left somatosensory cortex (lS1) The brain ROIs were selected based on literature supporting the importance of these regional involvements in pain and emotional processing (Chen et al., 2023; Gombaut and Holmes, 2022; Peng et al., 2018; Yang and Chang, 2019).

#### 2.3.1 Cognitive impairment

During the baseline visit, mild to moderate ADRD was determined using either the t-Moca (score of 16–26) or the MMSE (score of 16–23; Ahn et al., 2019). Half the participants took the MMSE (n = 20) and half took the t-MOCA (n = 20), due to COVID-19 provisions. To unify the different scales, we converted all scores to the MoCA-30 scale as shown in Table 2. The MoCA-30 (Julayanont et al., 2013; Nasreddine et al., 2005; Wittich et al., 2010) is a well-researched measure for cognitive dysfunction, demonstrates higher sensitivity and specificity for capturing MCI and early-onset Dementia, especially in patients with ADRD (Ciesielska et al., 2016; Freitas et al., 2013; Hsu et al., 2015; Trzepacz et al., 2015). It yields a total score with a maximum of 30 points, demonstrates strong specificity (82 %) and negative predictive value (84 %) (Gagnon et al., 2013), and was found to be feasible in telehealth contexts. Consistent with the approach of Jammula et al. (2022), the primary study conducted MoCA assessments via telehealth sessions using the t-MoCA test. To convert the MMSE scores to the MoCA-30, we used the Alzheimer's Dementia MMSE-MoCA-30 conversion table published by Roheger et al. (2022) and to convert the t- MoCA scores to the MoCA-30, we used a published conversion table by Katz et al. (2021). Using the cutoff score of 22 on the MoCA-30, we divided the participants into a “high cognitive function” (HCF) group (HCF; >22, n = 15) or a “low cognitive function” (LCF) group (LCF; ≤ 22, n = 25) (Islam et al., 2023). In our study population, 22 was both the median and the mode of the converted MoCA-30 scores (Ahn et al., 2019).

**Table 2:** Conversion from MMSE to MoCA-30 and t-MoCA to MoCA-30.

ID	MMSE	MMSE to MoCA-30	ID	MoCA-T	MoCA-T to MoCA-30	ID	MoCA-30 Final
101	21	15	122	19	25	101	15
102	24	19	123	16	22	102	19
103	24	19	124	18	24	103	19
104	20	14	125	10	14	104	14
105	28	22	126	17	23	105	22
106	25	20	127	11	15	106	20
107	9	4	128	17	23	107	4
108	30	26	129	16	22	108	26
109	5	2	130	19	25	109	2
110	21	15	131	19	25	110	15
111	28	22	132	19	25	111	22
112	24	19	133	17	23	112	19
113	29	23	134	16	22	113	23
114	20	14	135	17	23	114	14
115	21	15	136	19	25	115	15
116	19	13	137	16	22	116	13
117	29	23	138	16	22	117	23
118	21	15	139	18	24	118	15
119	21	15	140	17	23	119	15
120	26	21				120	21
121	26	21				121	21
						122	25
						123	22
						124	24
						125	14
						126	23
						127	15
						128	23
						129	22
						130	25
						131	25
						132	25
						133	23
						134	22
						135	23
						136	25
						137	22
						138	22
						139	24
						140	23

*Note.* MMSE to MoCA-30 Conversion (Roheger et al., 2022) and t-MoCA to MoCA-30 (Katz et al., 2021).

The NPI measured symptoms such as depression and apathy, with higher scores indicating greater severity (Cummings, 1994, 2020). Pain was assessed through self-reported NRS (scores 0–100), a validated tool for evaluating pain severity in dementia populations (Ngu et al., 2015).

#### 2.3.2 Functional near infrared spectroscopy (fNIRS)

**Fig. 1:**
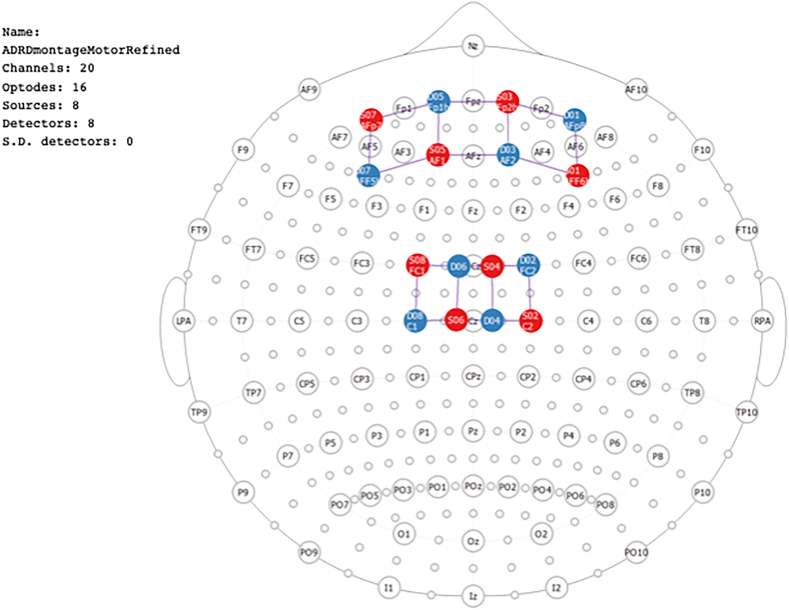
FNIRS probe design, with eight light sources (red circles) and eight light detectors (blue circles) forming 20 measurement channels (purple lines) represented over the 10-10 international system.

A continuous-wave multichannel fNIRS imaging system (LIGHTNIRS, Shimadzu, Kyoto, Japan) was employed to assess the brain hemodynamic changes using eight light sources, each emitting laser light at wavelengths of 780 and 830 nm, and eight detectors connected to comfortable headgear via optical fibers, the illumination and detection optodes were arranged in a geometrical layout that covered the prefrontal and somatosensory cortex regions bilaterally (Fig. 1). This configuration created 20 channels with a sampling rate of 13.3 Hz and an approximate average source-detector separation of 34.3 mm (sd = 8 mm). The fNIRS data were collected following thermal stimuli administered to the participant's right forearm at baseline, prior to any tDCS treatment. Optical fibers were secured to the participant's scalp using the fNIRS cap's grommets. The placement of illumination and detection optodes followed a geometric arrangement spanning both the PFC and S1 cortex regions on both sides, mirroring the locations examined in prior research (Ahn et al., 2019). Three sets of low-intensity thermal stimuli at or below the thermal pain threshold level were administered for 20 s each. Following each set, there was an approximate 30-s interval without stimulation. This protocol is frequently utilized in pain-related neuroimaging investigations due to the significance of thermal hyperalgesia in chronic pain. The Medoc TSA-II Neurosensory Analyzer, equipped with a 16 mm × 16 mm thermode, provided controlled thermal stimulation. Stimuli were kept below the participant's pain tolerance level, with a cutoff temperature of 50 °C, ensuring safety and comfort while enabling reliable imaging of pain-related cortical responses. Participants were notified they could withdraw their arm from the pain stimulation at any time.

### 2.4 Data processing

**Fig. 2:**
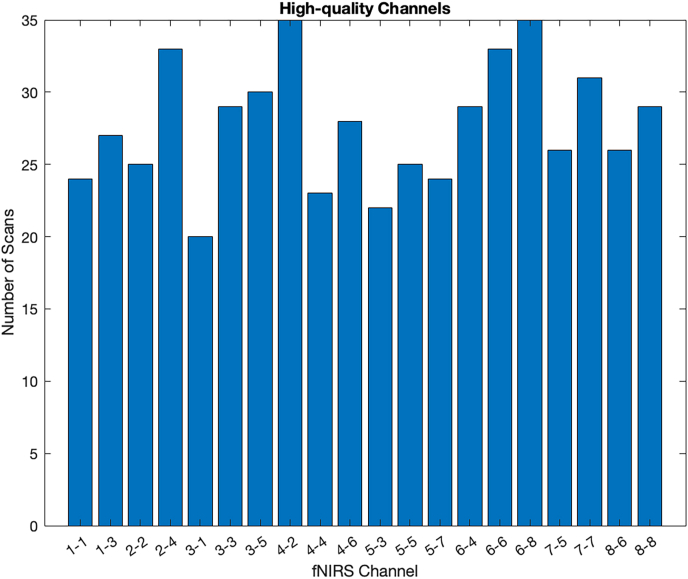
Number of fNIRS channels with high-quality surviving the QTNIRS evaluation.

The first step in the fNIRS pre-processing pipeline included the quality assessment of the data based on the cardiac pulsation using QTNIRS (Montero-Hernandez and Pollonini, 2020). Then, the surviving raw signals with evident presence of cardiac pulsation, as an indication of quality signals, were converted into optical densities (Fig. 2). Next, the data was band-pass filtered in the [0.01, 0.3] Hz range. The Temporal Derivative Distribution Repair (TDDR), a motion correction algorithm that does not require an artifact detection stage, was employed to correct artifacts like peaks and shifts in the baseline (Fishburn et al., 2019). Finally, the signals were resampled from 13.3 to 8 Hz and converted to oxy- (HbO) and deoxyhemoglobin (HbR) changes *(expressed in μM*) using the Modified Beer-Lambert Law (Cope et al., 1988; Delpy et al., 1988). The pre-processing steps were conducted using customized script using functions from NIRS Brain AnalyzIR and Homer3 toolboxes.

### 2.5 Brain regions-of-interest-based analysis

**Fig. 3:**
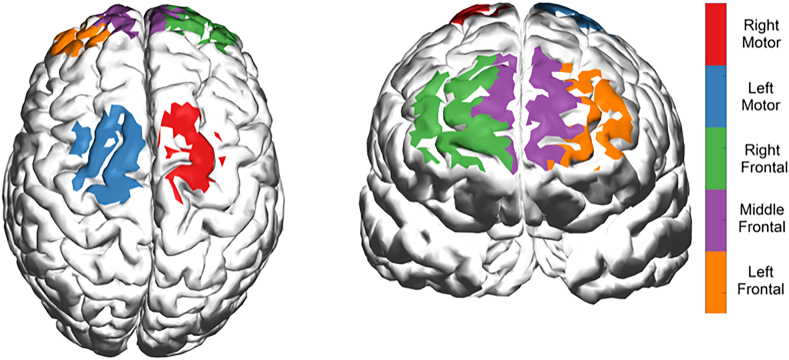
Five regions of interest defined covering left-, middle- and right-frontal and left- and right-motor areas.

**Fig. 4:**
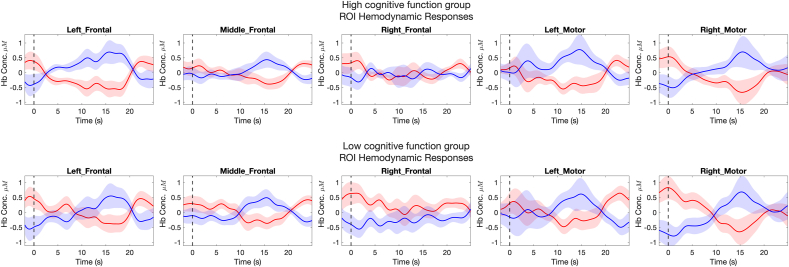
Group hemodynamic response functions (ROIs).

Five brain ROIs were defined to average the pre-processed data from channels covering neighboring regions. An ROI-based analysis usually involves obtaining averaged responses, which helps increase the signal-to-noise ratio. The five brain ROIs are shown in Fig. 3 and were defined as 1) right dorsolateral prefrontal cortex (rdlPFC) *with channels S01-D01, S01-D03, and S03-D01*, 2) medial prefrontal cortex (mPFC) *with channels S03-D03, S03-D05, S05-D03, and S05-D05*, 3) left dorsolateral prefrontal cortex (ldlPFC) *with channels S07-D05, S07-D07, and S05-D07,* 4) right somatosensory cortex (rS1) *with channels S02-D02, S02-D04, S04-D02, and S04-D04*, and 5) left somatosensory cortex (lS1) *with channels S06-D06, S06-D08, S08-D06, and S08-D08*. Once the ROIs were defined, HbO and HbR signals from channels were averaged according to the ROIs definition (Fig. 4)

For each ROI, we fitted the observed hemodynamic HbO and HbR responses to the expected response using a General Linear Model (Barker et al., 2013). The expected hemodynamic response was obtained by convolving a canonical response function (Santosa et al., 2018) with the stimulus train given by three trials with a duration of 20 s and an inter-stimulus interval of 30 s, each. Next, we obtained the coefficient beta (β), which measures the relative contribution of the pain stimulation to the fNIRS signal of a channel. When the value of beta is high for a particular channel, it means that the activation of the brain region covered by that channel closely follows the expected time course for that condition. A beta value close to 0 means that there is little to no relationship between the channel and the hypothesized time course. Also, beta values can be negative, meaning they are inversely related (activation decreases), also called deactivation. We conducted the analysis for each ROI across participants stratified by low and high cognitive function, MoCA=<22 and MoCA>22. Subsequent analysis focused on HbO signals given the higher signal-to-noise ratio than the HbR signal (Scholkmann et al., 2014). Finally, we used the algorithm autoregressive pre-whitening with iteratively reweighted least-squares (AR-IRLS) to solve the first-level GLM. The AR-IRLS-based solution has been shown to be sufficiently robust to the effects of physiology (serially correlated errors and colored noise) due to the capability of controlling type I (false-positive) even when a filtering process or motion artifact correction is not performed (Barker et al., 2013; Santosa et al., 2018).

## 3 Results

### 3.1 Correlation between hemodynamic responses and NPI measures

**Fig. 5:**
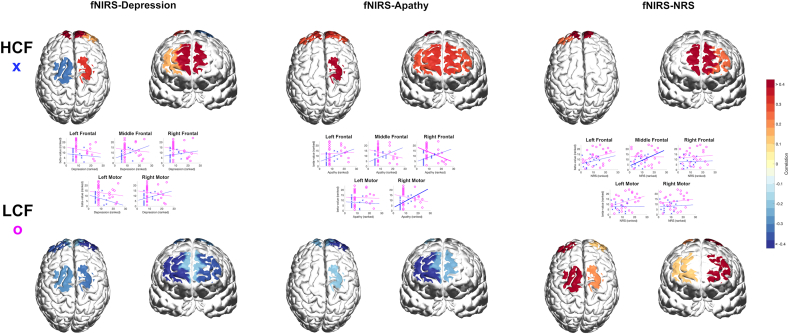
Depression, apathy, NRS, and fNIRS (beta-value) correlation. Horizontal and vertical axes represent the subjective metric and the beta-value after variable ranking, respectively. Thicker regression lines indicate statistically significant correlation.

We focused our analysis on two prevalent and clinically relevant neuropsychiatric symptoms in ADRD—depression and apathy—based on their known association with disease progression and functional decline. To investigate the relationship between the NPI variables and the HbO signals for each ROI, we measured the correlation between the beta coefficient (β) and depression and apathy. Using the ranked version of the NPI and beta values, we computed the correlation values using Spearman correlation (Fig. 5). The significance of the correlation values was computed using a T-test with the null case of zero correlation at the significance alpha value of 0.05 (Table 3).

**Table 3:** Correlation between fNIRS (beta values) and depression, apathy, and NRS variables.

fNIRS Variable	Subjective variable	Region of Interes (ROI)	Group	r	P-value
beta-value	Depression	Left_Frontal	LCF	−0.19	0.37
		Middle_Frontal		−0.11	0.60
		Right_Frontal		−0.22	0.31
		Left_Motor		−0.14	0.52
		Right_Motor		−0.16	0.45
	Apathy	Left_Frontal		0.06	0.79
		Middle_Frontal		−0.26	0.22
		**Right_Frontal**		**−0.42**	**0.04**
		Left_Motor		−0.04	0.87
		Right_Motor		−0.22	0.30
	NRS	Left_Frontal		0.31	0.14
		Middle_Frontal		−0.01	0.97
		Right_Frontal		0.09	0.68
		Left_Motor		0.33	0.11
		Right_Motor		0.14	0.50
	Depression	Left_Frontal	HCF	−0.10	0.73
		Middle_Frontal		0.36	0.18
		Right_Frontal		0.15	0.59
		Left_Motor		−0.29	0.29
		Right_Motor		0.27	0.33
	Apathy	Left_Frontal		0.36	0.19
		Middle_Frontal		0.41	0.13
		Right_Frontal		0.35	0.20
		Left_Motor		−0.05	0.85
		**Right_Motor**		**0.53**	**0.04**
	NRS	Left_Frontal		0.28	0.32
		**Middle_Frontal**		**0.52**	**0.04**
		Right_Frontal		0.03	0.92
		Left_Motor		−0.01	0.97
		Right_Motor		0.07	0.81

#### 3.1.1 Low cognitive function group

No significant correlations between beta values from HbO and depression across all brain ROIs were observed. Table 3 shows r and p-values associated with each brain region and variable measured. Apathy was inversely correlated with values for mPFC and rdlPFC, and left- and right-S1 ROIs. However, only the rdlPFC region achieved statistical significance (r = −0.42, p-value = 0.04). No significant correlations were found for the fNIRS-NRS pair.

#### 3.1.2 High cognitive function group

For depression, we observed positive correlation values for mPFC and, rdlPFC, and rS1 ROIs, while ldlPFC and lS1 showed negative correlations. However, none of the beta-depression correlation values were statistically significant for the high cognitive function group. Regarding beta-apathy correlation, all except one (lS1) ROI exhibited positive correlation values. Further, a significant correlation value was found for the rS1region (r = 0.53, p-value = 0.04). The correlation values for both groups can be found in Table 3.

#### 3.1.3 Group comparisons

In terms of group comparison, we observed opposite correlation results between the low cognitive function (LCF) group and high cognitive function (HCF) group in mPFC and rdlPFC and left-and right-S1ROIs for both depression and apathy correlations. This can be interpreted as higher scores for depression and apathy leading to a lower observed hemodynamic response in mPFC and rdlPFC, as well as rS1 regions for the group with lower cognitive function. In contrast, high depression and apathy scores evoked increased hemodynamic activity for the high cognitive function group in the same areas. Regarding NRS, a significant correlation was found in the mPFC (r = 0.52, p-value = 0.04).

## 4 Discussion and future direction

In this study, we analyzed fNIRS data from five brain RIOs upon application of subthreshold thermal pain to the right forearm of 40 individuals with confirmed ADRD and chronic osteoarthritic knee pain, prior to tDCS intervenion. As an exploratory study, we examined correlations between changes in oxyhemoglobin and self-reported symptoms of depression, apathy, and pain across two groups – low cognitive function and high cognitive function. ADRD is a progressive disorder causing neuronal degeneration in the brain, often accompanied by depression and symptoms of apathy. As this degeneration process occurs, cognitive and verbal functions become more impacted, making it difficult for the patient to consistently and accurately self-report pain and psychiatric symptoms. The results of this pilot study show hemodynamic differences across the LCF and HCF groups for each of the three variables measured in several brain ROIs, providing preliminary evidence that fNIRS can detect unique biomarkers related to pain and specific neuropsychiatric symptoms, particularly depression and apathy in individuals with more severe cognitive decline. Understanding the relationship between cerebral metabolic changes in individuals with mild to moderate cognitive dysfunctions and chronic pain and neuropsychiatric symptoms provides critical biological and clinical insights into disease mechanisms, symptom severity, and treatment responses, improving clinical diagnostics, symptom monitoring, and interventions.

### 4.1 Depression

No statistically significant correlations between oxyhemoglobin (HbO) and depression scores were found in either the LCF or HCF group. However, directional trends suggest negative correlations in the LCF group, possibly indicating decreased activity in emotional regulation regions, and positive correlations in the HCF group, reflecting intact neural pathways or heightened emotional awareness, which align with what we would expect given the degenerative process of ADRD.

### 4.2 Apathy

The LCF group showed significant deactivation in the rdlPFC (r = −0.42, p = .04), consistent with prior studies linking apathy to decreased cortical activation in advanced cognitive decline (Braine and Georges, 2023; Gombaut and Holmes, 2022; Leite et al., 2017; Moretti and Signori, 2016). The HCF group exhibited significant activity in rS1 (r = 0.53, p = .04). Given that the thermal evoked pain stimulus was applied to the right forearm, we would expect to see a positive correlation contralateral to the stimulus (left hemisphere) in cognitively intact individuals. Although nonsignificant, a strong negative correlation was observed in the left S1 cortex, suggesting compensatory mechanisms may be at play. When the brain is injured or neuropathways are disrupted, it learns to adapt as a protective measure. Research indicates compensatory mechanisms in the brain occur early in the neurodegenerative process, even before noticeable symptoms of dementia occur (Bobkova and Vorobyov, 2015; Ferrari et al., 2016), creating potential discrepancies between cognitive and functional performance and brain biomarkers.

For both depression and apathy, the HCF group self-reported greater symptoms of depression and apathy than the LCF group. Cognitive function may be associated with self-awareness and accuracy of self-identification of emotional pain, which should be considered when reviewing the findings, as the participants were not clinically assessed for depression or apathy, limiting our understanding of the scores.

### 4.3 Pain

A significant positive correlation between HbO and pain scores was observed in mPFC for the HCF group (r = 0.52, p = .04). This region plays a significant role in processing the emotional and cognitive mechanism of pain, particularly an inhibitory role of both sensory and affective pain (Kummer et al., 2020). Our results reflect preserved pain processing in the HCF group. Weak and nonsignificant correlations in the LCF group may indicate impaired pain perception or pain threshold reporting accuracy. For those in the LCF group, it is possible that their ability to sense or articulate pain accurately may be compromised, leading to less accurate subthreshold pain level identification.

Using neuroimaging to identify hemodynamic changes in brain ROIs at different stages of cognitive function may provide critical insight, aiding in earlier identification and treatment for dementia. Our findings align with current studies indicating less activation in the PFC with progressed ADRD (Mei et al., 2023; Robba et al., 2023), further making the case for fNIRS as a viable tool for quantifying pain and NPS in the brain.

### 4.4 Limitations and future directions

These results should be interpreted within the parameters of several limitations.

#### 4.4.1 Sample

The small, homogenous sample (n = 40; 72 % female, 90 % White) limits generalizability. Additionally, the discrete responses on the NPI limit variability in our analysis for such a small sample. Future studies should include larger, diverse populations and stratify results by race/ethnicity.

#### 4.4.2 Study design

The exploratory approach identifies patterns but may weaken p-value strength. Only self-reported NPS and pain data were analyzed for this study. Future studies should include a clinical assessment to supplement the self-report data and differentiate types of dementia, as different patterns of degeneration emerge for different subtypes of dementia. This is especially important for individuals with varying levels of cognitive decline, potentially affecting their abilities to accurately self-report symptoms. Finally, as the study examined only two neuropsychiatric symptoms—specifically, depression and apathy—the findings should not be generalized to the full range of neuropsychiatric symptoms observed in ADRD.

#### 4.4.3 Confounding variables

This study did not adjust for potential confounding factors such as medication use, caregiver-reported symptoms, or other contextual variables that may influence neuropsychiatric symptom presentation and pain perception. Although individualized pain thresholds were used to determine subthreshold stimulation levels, additional analyses accounting for these confounders will be important in future studies with larger samples.

#### 4.4.4 fNIRS

While fNIRS imaging shows promise for objectively measuring neuronal hemodynamic activity in certain brain regions, it is less effective for deeper brain regions (e.g., ventral prefrontal cortex). To mitigate this limitation, future studies should explore brain hemodynamic changes using high-density fNIRS devices, which offer a denser coverage of brain regions, allowing for brain image reconstruction comparable to fMRI (Vidal-Rosas et al., 2023). Additionally, advanced connectivity methods to map brain networks may contribute to identifying causal relationships among brain regions (Arab et al., 2025). This study highlights the potential of fNIRS to detect distinct hemodynamic patterns associated with pain and neuropsychiatric symptoms in individuals with ADRD, varying by cognitive function level. While significant correlations were limited in this study, the observed trends and activity differences provide valuable insights into the interplay between cognitive decline, emotional processing, and pain perception. The findings underscore the importance of expanding research to include larger, more diverse samples and integrating clinical assessments to validate self-reported data. Moreover, advancing fNIRS methodologies to capture deeper brain activity and connectivity patterns could further enhance its utility. These efforts could pave the way for improved diagnosis, monitoring, and intervention strategies for managing ADRD-related symptoms. While this study focused solely on depressive symptoms and apathy, the findings may inform future research examining a broader range of neuropsychiatric symptoms in ADRD.

## CRediT authorship contribution statement

**Allison J. Huff:** Writing – review & editing, Writing – original draft, Methodology, Data curation, Conceptualization. **Juyoung Park:** Writing – review & editing, Writing – original draft, Conceptualization. **Samuel Montero-Hernandez:** Writing – review & editing, Writing – original draft, Methodology, Formal analysis, Conceptualization. **Lindsey Park:** Writing – review & editing, Project administration, Data curation. **Chiyoung Lee:** Writing – review & editing, Conceptualization. **Luca Pollonini:** Writing – review & editing, Investigation, Formal analysis, Data curation, Conceptualization. **Hyochol Ahn:** Writing – review & editing, Supervision, Project administration, Methodology, Investigation, Funding acquisition, Formal analysis, Conceptualization.

## Declaration of competing interest

The authors declare the following financial interests/personal relationships which may be considered as potential competing interests: Hyochol Ahn reports financial support was provided by 10.13039/100000056National Institute of Nursing Research. If there are other authors, they declare that they have no known competing financial interests or personal relationships that could have appeared to influence the work reported in this paper.

## Data Availability

Data will be made available on request.

## References

[bib1] Achterberg W., Lautenbacher S., Husebo B., Erdal A., Herr K. (2019). Pain in dementia. Pain reports.

[bib2] Ahn H., Zhong C., Miao H., Chaoul A., Park L., Yen I.H., Vila M.A., Sorkpor S., Abdi S. (2019). Efficacy of combining home-based transcranial direct current stimulation with mindfulness-based meditation for pain in older adults with knee osteoarthritis: a randomized controlled pilot study. J. Clin. Neurosci.: Official Journal of the Neurosurgical Society of Australasia.

[bib3] Alzheimer’s Association (2024). Alzheimer's disease facts and figures. Alzheimer's Dement..

[bib4] Arab F., Ghassami A., Jamalabadi H., Peters M., Nozari E. (2025). Whole-brain causal discovery using fMRI. Network Neuroscience.

[bib5] Atri A., Dickerson B., Atri A. (2014). Dementia: Comprehensive principles and Practice.

[bib6] Barker J.W., Aarabi A., Huppert T. (2013). Autoregressive model based algorithm for correcting motion and serially correlated errors in fNIRS. Biomed. Opt. Express.

[bib7] Bobkova N., Vorobyov V. (2015). The brain compensatory mechanisms and Alzheimer's disease progression: a new protective strategy. Neural regeneration research.

[bib8] Bornier N., Mulliez A., Chenaf C., Elyn A., Teixeira S., Authier N., Bertin C., Kerckhove N. (2023). Chronic pain is a risk factor for incident Alzheimer's disease: a nationwide propensity-matched cohort using administrative data. Front. Aging Neurosci..

[bib9] Braine A., Georges F. (2023). Emotion in action: when emotions meet motor circuits. Neurosci. Biobehav. Rev..

[bib10] Brayne C., Miller B. (2017). Dementia and aging populations—a global priority for contextualized research and health policy. PLoS Med..

[bib11] Breivik H., Borchgrevink P.C., Allen S.M., Rosseland L.A., Romundstad L., Hals E.K., Kvarstein G., Stubhaug A. (2008). Assessment of pain. Br. J. Anaesth..

[bib12] Cao S., Fisher D.W., Yu T., Dong H. (2019). The link between chronic pain and Alzheimer's disease. J. Neuroinflammation.

[bib13] Centers for Disease Control and Prevention (CDC) (2019).

[bib14] Chen J., Wang X., Xu Z. (2023). The relationship between chronic pain and cognitive impairment in the elderly: a review of current evidence. J. Pain Res..

[bib15] Chen W.L., Wagner J., Heugel N., Sugar J., Lee Y.W., Conant L., Malloy M., Heffernan J., Quirk B., Zinos A., Beardsley S.A., Prost R., Whelan H.T. (2020). Functional near-infrared spectroscopy and its clinical application in the field of neuroscience: advances and future directions. Front. Neurosci..

[bib16] Ciesielska N., Sokołowski R., Mazur E., Podhorecka M., Polak-Szabela A., Kędziora- Kornatowska K. (2016). Is the Montreal Cognitive Assessment (MoCA) test better suited than the Mini-Mental State Examination (MMSE) in mild cognitive impairment (MCI) detection among people aged over 60? Meta-analysis. Psychiatr. Pol..

[bib17] Cole L.J., Farrell M.J., Gibson S.J., Egan G.F. (2010). Age-related differences in pain sensitivity and regional brain activity evoked by noxious pressure. Neurobiol. Aging.

[bib18] Cope M., Delpy D.T., Reynolds E.O., Wray S., Wyatt J., van der Zee P. (1988). Methods of quantitating cerebral near infrared spectroscopy data. Adv. Exp. Med. Biol..

[bib19] Cravello L., Di Santo S., Varrassi G., Benincasa D., Marchettini P., de Tommaso M., Shofany J., Assogna F., Perotta D., Palmer K., Paladini A., di Iulio F., Caltagirone C. (2019). Chronic pain in the elderly with cognitive decline: a narrative review. Pain and therapy.

[bib20] Crosson B., Ford A., McGregor K.M., Meinzer M., Cheshkov S., Li X., Walker-Batson D., Briggs R.W. (2010). Functional imaging and related techniques: an introduction for rehabilitation researchers. J. Rehabil. Res. Dev..

[bib21] Cummings J.L. (2020). The neuropsychiatric inventory: development and applications. J. Geriatr. Psychiatr. Neurol..

[bib22] Cummings J.L., Mega M., Gray K., Rosenberg-Thompson S., Carusi D.A., Gornbein J. (1994). The Neuropsychiatric Inventory: comprehensive assessment of psychopathology in dementia. Neurology.

[bib23] Delpy D.T., Cope M., van der Zee P., Arridge S., Wray S., Wyatt J. (1988). Estimation of optical pathlength through tissue from direct time of flight measurement. Phys. Med. Biol..

[bib24] Falcon C., Montesinos P., Václavů L., Kassinopoulos M. (2024). Time-encoded ASL reveals lower cerebral blood flow in the early AD continuum. Alzheimer's Dement..

[bib25] Fernandez-Rojas R., Huang X., Ou K.L. (2017). Toward a functional near-infrared spectroscopy-based monitoring of pain assessment for nonverbal patients. J. Biomed. Opt..

[bib27] Ferrari F., Vecchio F., Vollero L., Guerra A., Petrichella S., Ponzo D., Määtta S., Mervaala E., Könönen M., Ursini F., Pasqualetti P., Iannello G., Rossini P.M., Di Lazzaro V. (2016). Sensorimotor cortex excitability and connectivity in Alzheimer's disease: a TMS-EEG Co-registration study. Hum. Brain Mapp..

[bib28] Fishburn F.A., Ludlum R.S., Vaidya C.J., Medvedev A.V. (2019). Temporal derivative distribution repair (tddr): a motion correction method for fNIRS. Neuroimage.

[bib29] Freitas S., Simões M.R., Alves L., Santana I. (2013). Montreal cognitive assessment. Alzheimer Dis. Assoc. Disord..

[bib30] Gagnon G., Hansen K.T., Woolmore-Goodwin S., Gutmanis I., Wells J., Borrie M., Fogarty J. (2013). Correcting the MoCA for education: effect on sensitivity. The Canadian journal of neurological sciences. Le journal canadien des sciences neurologiques.

[bib31] Gombaut C., Holmes S.A. (2022). Sensorimotor integration and pain perception: mechanisms integrating nociceptive processing. A systematic review and ALE-meta analysis. Front. Integr. Neurosci..

[bib32] Hsu J.L., Fan Y.C., Huang Y.L. (2015). Improved predictive ability of the Montreal Cognitive Assessment for diagnosing dementia in a community-based study. Alz Res Therapy.

[bib33] Islam N., Hashem R., Gad M., Brown A., Levis B., Renoux C., Thombs B.D., McInnes M.D. (2023). Accuracy of the Montreal Cognitive Assessment tool for detecting mild cognitive impairment: a systematic review and meta-analysis. Alzheimer's & dementia : the journal of the Alzheimer's Association.

[bib34] Jammula V., Rogers J.L., Vera E., Christ A., Leeper H.E., Acquaye A., Briceno N., Choi A., Grajkowska E., Levine J.E., Lindsley M., Reyes J., Roche K.N., Timmer M., Boris L., Burton E., Lollo N., Panzer M., Smith-Cohn M.A., Penas-Prado M. (2022). The Montreal Cognitive Assessment (MoCA) in neuro-oncology: a pilot study of feasibility and utility in telehealth and in-person clinical assessments. Neuro-Oncol. Pract..

[bib35] Julayanont P., Phillips N., Chertkow H., Nasreddine Z.S., Larner A.J. (2013). Cognitive Screening Instruments: A Practical Approach.

[bib36] Karunakaran K.D., Peng K., Berry D., Green S., Labadie R., Kussman B., Borsook D. (2021). NIRS measures in pain and analgesia: fundamentals, features, and function. Neurosci. Biobehav. Rev..

[bib37] Katz M.J., Wang C., Nester C.O., Derby C.A., Zimmerman M.E., Lipton R.B., Sliwinski M.J., Rabin L.A. (2021). T-MoCA: a valid phone screen for cognitive impairment in diverse community samples. Alzheimer’s & Dementia.

[bib38] Khan M.U., Sousani M., Hirachan N., Joseph C., Ghahramani M., Chetty G., Goecke R., Fernandez-Rojas R. (2024). Multilevel pain assessment with functional near-infrared spectroscopy: evaluating Δ*HBO*_2_ and Δ*HHB* measures for comprehensive analysis. Sensors.

[bib39] Kim D., Chae Y., Park H.J., Lee I.S. (2021). Effects of chronic pain treatment on altered functional and metabolic activities in the brain: a systematic review and meta-analysis of functional neuroimaging studies. Front. Neurosci..

[bib40] Koyanagi M., Yamada M., Higashi T., Mitsunaga W., Moriuchi T., Tsujihata M. (2021). The usefulness of functional near-infrared spectroscopy for the assessment of post-stroke depression. Front. Hum. Neurosci..

[bib41] Kummer K.K., Mitrić M., Kalpachidou T., Kress M. (2020). The medial prefrontal cortex as a central hub for mental comorbidities associated with chronic pain. Int. J. Mol. Sci..

[bib42] Kuruvilla M.S., Green J.R., Ayaz H., Murman D.L. (2013). Neural correlates of cognitive decline in ALS: an fNIRS study of the prefrontal cortex. Cognit. Neurosci..

[bib43] Langa K.M. (2015). Is the risk of Alzheimer's disease and dementia declining?. Alzheimers Res. Ther..

[bib44] Lee T.L., Guo L., Chan A.S. (2024). fNIRS as a biomarker for individuals with subjective memory complaints and MCI. Alzheimer's & dementia : the journal of the Alzheimer's Association.

[bib45] Leite J., Carvalho S., Battistella L.R., Caumo W., Fregni F. (2017). Editorial: the role of primary motor cortex as a marker and modulator of pain control and emotional-affective processing. Front. Hum. Neurosci..

[bib46] Lichtner V., Dowding D., Esterhuizen P., Closs S.J., Long A.F., Corbett A., Briggs M. (2014). Pain assessment for people with dementia: a systematic review of systematic reviews of pain assessment tools. BMC Geriatr..

[bib47] Mather M., Scommegna P. (2024). Fact sheet: aging in the United States. Population Bureau Reference.

[bib48] Mei X., Zou C.J., Hu J., Liu X.L., Zheng C.Y., Zhou D.S. (2023). Functional near- infrared spectroscopy in elderly patients with four types of dementia. World J. Psychiatr..

[bib49] Montero-Hernandez S., Pollonini L., Park L., Martorella G., Miao H., Mathis K.B., Ahn H. (2023). Self-administered transcranial direct current stimulation treatment of knee osteoarthritis alters pain-related fNIRS connectivity networks. Neurophotonics.

[bib50] Montero-Hernandez S., Pollonini L. (2020). *OSA Technical Digest* Biophotonics Congress: Biomedical Optics 2020 (Translational, Microscopy, OCT, OTS, BRAIN).

[bib51] Moretti R., Signori R. (2016). Neural correlates for apathy: frontal-prefrontal and parietal cortical- subcortical circuits. Front. Aging Neurosci..

[bib52] Nasreddine Z.S., Phillips N.A., Bédirian V., Charbonneau S., Whitehead V., Collin I., Cummings J.L., Chertkow H. (2005). The Montreal Cognitive Assessment, MoCA: a brief screening tool for mild cognitive impairment. J. Am. Geriatr. Soc..

[bib53] Ng K.P., Chiew H.J., Rosa-Neto P., Kandiah N., Ismail Z., Gauthier S. (2019). Brain metabolic dysfunction in early neuropsychiatric symptoms of dementia. Front. Pharmacol..

[bib54] Ngu S.S., Tan M.P., Subramanian P., Abdul Rahman R., Kamaruzzaman S., Chin A.V., Tan K.M., Poi P.J. (2015). Pain assessment using self-reported, nurse-reported, and observational pain assessment tools among older individuals with cognitive impairment. Pain Manag. Nurs. : official journal of the American Society of Pain Management Nurses.

[bib55] Nguyen T., Behrens M., Broscheid K.C., Bielitzki R., Weber S., Libnow S., Malczewski V., Baldauf L., Milberger X., Jassmann L., Wustmann A., Meiler K., Drange S., Franke J., Schega L. (2023). Associations between gait performance and pain intensity, psychosocial factors, executive functions as well as prefrontal cortex activity in chronic low back pain patients: a cross-sectional fNIRS study. Front. Med..

[bib56] Ong W.Y., Stohler C.S., Herr D.R. (2019). Role of the prefrontal cortex in pain processing. Mol. Neurobiol..

[bib57] Peng K., Yücel M.A., Aasted C.M., Steele S.C., Boas D.A., Borsook D., Becerra L. (2018). Using prerecorded hemodynamic response functions in detecting prefrontal pain response: a functional near-infrared spectroscopy study. Neurophotonics.

[bib58] Pless A., Ware D., Saggu S., Rehman H., Morgan J., Wang Q. (2023). Understanding neuropsychiatric symptoms in Alzheimer's disease: challenges and advances in diagnosis and treatment. Front. Neurosci..

[bib59] Pollonini L., Miao H., Ahn H. (2020). Longitudinal effect of transcranial direct current stimulation on knee osteoarthritis patients measured by functional infrared spectroscopy: a pilot study. Neurophotonics.

[bib60] Quaresima V., Ferrari M. (2019). Functional near-infrared spectroscopy (fNIRS) for assessing cerebral cortex function during human behavior in natural/social situations: a concise review. Organ. Res. Methods.

[bib61] Robba C., Battaglini D., Rasulo F., Lobo F.A., Matta B. (2023). The importance of monitoring cerebral oxygenation in non brain injured patients. J. Clin. Monit. Comput..

[bib62] Roheger M., Xu H., Hoang M.T., Eriksdotter M., Garcia-Ptacek S. (2022). Conversion between the mini-mental state examination and the Montreal cognitive assessment for patients with different forms of dementia. J. Am. Med. Dir. Assoc..

[bib63] Santosa H., Zhai X., Fishburn F., Huppert T. (2018). The NIRS brain AnalyzIR toolbox. Algorithms.

[bib64] Scholkmann F., Kleiser S., Metz A.J., Zimmermann R., Mata Pavia J., Wolf U., Wolf M. (2014). A review on continuous wave functional near-infrared spectroscopy and imaging instrumentation and methodology. Neuroimage.

[bib65] Smits L.L., van Harten A.C., Pijnenburg Y.A.L., Koedam E.L.G.E., Bouwman F.H., Sistermans N. (2015). Trajectories of cognitive decline in different types of dementia. Psychol. Med..

[bib66] Soucy J.P., Bartha R., Bocti C., Borrie M., Burhan A.M., Laforce R., Rosa-Neto P. (2013). Clinical applications of neuroimaging in patients with alzheimer's disease: a review from the fourth Canadian consensus conference on the diagnosis and treatment of dementia 2012. Alzheimers Res. Ther..

[bib67] Tori K., Kalligeros M., Nanda A., Shehadeh F., van Aalst R., Chit A., Mylonakis E. (2020). Association between dementia and psychiatric disorders in long-term care residents: an observational clinical study. Medicine.

[bib68] Trzepacz P.T., Hochstetler H., Wang S., Walker B., Saykin A.J., Alzheimer’s Disease Neuroimaging Initiative (2015). Relationship between the Montreal Cognitive Assessment and Mini-mental State Examination for assessment of mild cognitive impairment in older adults. BMC Geriatr..

[bib69] Vidal-Rosas E.E., von Luhman A.A., Pinti P., Cooper R.J. (2023). Wearable, high-density fNIRS and diffuse optical tomography technologies: a perspective. Neurophoton.

[bib70] Wimo A., Seeher K., Cataldi R., Cyhlarova E., Dielemann J.L., Frisell O., Guerchet M., Jönsson L., Malaha A.K., Nichols E., Pedroza P., Prince M., Knapp M., Dua T. (2023). The worldwide costs of dementia in 2019. Alzheimer's & dementia : the journal of the Alzheimer's Association.

[bib71] Wittich W., Phillips N., Nasreddine Z., Chertkow H. (2010). Sensitivity and specificity of the Montreal cognitive assessment modified for individuals who are visually impaired. J. Vis. Impair. Blind. (JVIB).

[bib72] Xie L., Liu Y., Gao Y., Zhou J. (2024). Functional Near-Infrared Spectroscopy in neurodegenerative disease: a review. Front. Neurosci..

[bib73] Yang S., Chang M.C. (2019). Chronic pain: structural and functional changes in brain structures and associated negative affective states. Int. J. Mol. Sci..

[bib74] Zhang Z., Gewandter J.S., Geha P. (2022). Brain imaging biomarkers for chronic pain. Front. Neurol..

